# Editorial

**Published:** 1868-03-15

**Authors:** 


### EDITORIAL.

**Rush Medical College.**

*Programme of Spring Course, commencing Monday, March 2nd, 1868:*

| | | |
|---|---|---|
| Dr. Holmes, Mondays and Thursdays, | 2 | o'clock. |
| Prof. Blaney, " " " | 4 | " |
| Dr. Parkes, Tuesdays and Fridays, | 2 | " |
| Dr. Fenn, " " " | 10 | " |
| Dr. Owens, Wednesdays and Saturdays, | 10 | " |
| Dr. Lyman, Mondays and Wednesdays, | 11 | " |

Dr. Marsh, every day, 3 o'clock. *Dispensary.*

Prof. Freer, Mondays and Thursdays, 10 o'clock.

Prof. Gunn, Surgical Clinic, every Saturday at 2 o'clock.

**Our Paris Article.**

Owing to the non-arrival from Paris of the remaining pages of the article being translated for the JOURNAL by Dr. Hay, we are reluctantly compelled to send our forms to press without waiting any longer for them. The remainder of the article will appear as soon as received.

**Our Philadelphia Letter.**

Our Philadelphia letter, in the last number, by some unaccountable mistake, was not placed under its appropriate heading. Readers, however, will readily recognize the initials "E. R. H.," and thus give the proper location of the "Clinical Cases."

**Nasal Douche.**

A very convenient little apparatus is the *Nasal Douche*—see advertising pages. Easily managed, and not liable to get out of repair, we have found by experience that it affords a very successful means of treating various affections of the nose. The attention of those of our readers who have not tried it, is especially directed to the article.

***Back Numbers.***

Back numbers for 1867 can now be supplied to those of our subscribers who wish to complete their volumes.

***To our Subscribers.***

Subscribers who have not received their copy of the "Endoscope," as per our prospectus of last December, by notifying us will have it promptly forwarded. We shall take it as an especial favor to be notified of any failure to receive the JOURNAL on time, as our arrangements contemplate no delay in issue or failure in mailing the numbers.

